# The Effects of Using Virtual Reality on Thai Word Order Learning

**DOI:** 10.3390/brainsci13030517

**Published:** 2023-03-20

**Authors:** Nitiwat Watthanapas, Yung-Wei Hao, Jian-Hong Ye, Jon-Chao Hong, Jhen-Ni Ye

**Affiliations:** 1 BA Program in Southeast Asian Languages and Cultures, National Chengchi University, Taipei City 116, Taiwan; 2 Graduate Institute of Curriculum and Instruction, National Taiwan Normal University, Taipei City 106, Taiwan; 3 Faculty of Education, Beijing Normal University, Beijing 100875, China; 4 National Institute of Vocational Education, Beijing Normal University, Beijing 100875, China; 5 Department of Industrial Education, National Taiwan Normal University, Taipei City 106, Taiwan; 6 Chinese Language and Technology Center, National Taiwan Normal University, Taipei City 106, Taiwan; 7 Graduate Institute of Technological & Vocational Education, National Taipei University of Technology, Taipei City 106, Taiwan

**Keywords:** cognitive theory of multimedia learning, Human-Computer Interaction (HCI), learning retention, task value, Thai language learning, theories of embodied cognition, virtual reality, word order learning

## Abstract

Thai has its own unique spelling system and grammatical rules. Its word order is quite different from that of Mandarin and English, thus making it more difficult for students in Taiwan to learn. Past studies also point out that learning word order is one of the most difficult aspects when learning foreign languages. As science and technology advance, emerging technologies have been widely applied in foreign language learning. This research aims to explore the effect of using a multi-language VR learning assessment system on assisting Thai learners to learn grammatical word order, and to investigate the correlates between Thai self-efficacy, Thai language anxiety, word order learning retention, and task value of VR learning. In order to accomplish this purpose, we invited Thai learners who took Thai courses in the continuing education division of a national university in northern Taiwan to participate in a 5-week teaching experiment, during which the participants were asked to practice Thai word order for 20 min. They were administered a questionnaire to fill out after five weeks of practice and were tested for retention one month after the experiment. A total of 84 valid questionnaires were collected, with an effective return rate of 93.3%. Of the respondents, 30 were male (35.7%), and 54 were female (64.3%). The data were subjected to item analysis, reliability and validity analysis, and then underwent PLS-SEM for research model validation. The results revealed that: (1) Thai language self-efficacy was positively related to learning retention and task value; (2) Thai language anxiety was negatively related to learning retention and task value; (3) Learning retention was positively related to the task value of learning and continuous usage intention.

## 1. Introduction

Thai writing is a spelling system with its own distinctive alphabet [1]. A meaningful Thai word may have one single syllable or may be composed of two or more syllables. The alphabet and phonetic system are quite complex compared to English, and normally it takes considerable time for learners to memorize the complicated and puzzling combinations of consonants, vowels, and tone marks. Also, in Thai writing, there are generally no separators such as dots, commas, or other punctuation marks, and not even spaces to separate words in a sentence. Therefore, when reading in Thai, people have to segment words using cues other than spaces [2]. Knowing these distinctions can be a challenge for non-native speakers, not to mention learning the grammatically correct word order, which is crucial to the comprehension of Thai.

The challenges faced by Thai learners in Taiwan, be they elementary, high school, college students, or students of extension education or cram school, are generally the same. One of the reasons is the lack of a naturalistic setting, and as such, learners tend to compose sentences with Mandarin grammar and structure first and try to translate them into Thai word by word. In this way, they have a high chance of stumbling over grammar rules, especially over word order, which is completely different between the two languages. Therefore, frequent practice of the word order in Thai is of major importance. As science and technology advance, emerging technologies have been gradually applied in foreign language learning. With the purpose of encouraging learners to do more exercise and thereby helping to enhance their proficiency in Thai word order, this research aimed to explore the effect of adopting new technology for Thai word order learning.

Successful language learning largely relies on a variety of complex factors [3]. In recent decades, there has been a large body of research and pedagogical experimentation on using technology in foreign language education [4], and educational technology has been confirmed to have a substantial and positive effect on teaching and learning [5]. Today, in the age of information and technology, language learning settings have great influences on adult language learners’ study and understanding [6]. Past studies revealed that language learners learning with multimedia practices perform better in vocabulary learning than with paper-based materials [7]. Many educators or scholars suggest using multimedia in foreign language teaching to help reinforce language learning [8], and among these tools, VR (virtual reality) can be a potential tool for transformative learning and teaching [9].

As technological and scientific innovations are outpacing the speed of human learning, the need for innovative teaching techniques and environments is becoming increasingly important [10]. In the beginning, VR systems were invented mainly for entertainment and gaming, but many studies have demonstrated that VR environments have great importance and potential to serve as a pedagogical tool to aid and improve learning in the educational process [11]. Other research has also reported that VR systems could provide learning outcomes the same as or better than textbook assignments, and would bring great benefits to teaching activities [12]. The utilization of VR technology may, thereby, bring meaningful learning experiences to learners.

Immersive learning through VR is one of the main trends in education. Immersive learning provides better learning experiences for learners through simulated or artificial environments [13]. Virtual experiences can help expand learning dimensions [14], as immersive environments can facilitate learning, training, and other activities in a motivating and participatory way [15]. Additionally, immersive technology has the potential to overcome physical limitations and bring on-site field experiences into the classroom, and immersive and interactive VR simulations provide a high degree of immersion and enjoyment as well as significant positive learning outcomes [16]. When used in foreign language learning, a virtual environment can provide learners with a diverse and dynamic setting for vocabulary learning, in which learners can navigate on their own and thereby attain better autonomy and cognitive engagement than in traditional classrooms, and the quality of interaction during practice is improved accordingly [17]. Hence, an immersive learning environment in VR will create a better learner-centered learning field.

While learning foreign languages often brings enormous challenges for adult learners, many platforms have been developed for effective foreign language teaching. Among them, immersive VR is considered a particularly effective foreign language learning platform [18]. Therefore, in this research, we used a multi-language VR learning assessment system to conduct a teaching experiment on learning Thai sentence structures. We attempted to explore the interplay between Thai self-efficacy, language anxiety, word order learning retention, and task value in a VR learning environment.

## 2. Research Methods

### 2.1. Theoretical Framework

With the above-mentioned purposes, this research adopted the cognitive theory of multimedia learning (CTML) and learning based on theories of embodied cognition (TEC) to examine the effects of applying virtual reality in Thai word order learning.

Embodied learning refers to knowledge construction in a physical and mental action that joins body and mind [19], and embodiment theory argues that knowledge is obtained through the sensorimotor system [20]. Embodied learning also contributes to the construction of contemporary teaching and learning theories, which highlight the utilization of the body in acquiring knowledge during the educational process [21]. These theories are being applied in the design of learning environments, and new technologies have been designed to be able to respond to learners’ physical activities [22], which have been shown to enhance memory performance when incorporated into learning [23]. The thought of experimenting with the effect of applying VR on Thai word order learning was based on the concept of embodied learning.

According to Mayer, the cognitive theory of multimedia learning (CTML) describes the cognitive process involved in multimedia learning and with prior knowledge activated from long-term memory [24]. Multimedia learning refers to learning through combining a set of words and pictures, as well as a wide range of multimedia materials and tools provided [25], which can be still images or animations [26]. In the cognitive theory of multimedia learning, a piece of instructional information presented to the learner causes the learner’s cognitive processing, thereby producing learning outcomes [27]. The immersion principle, however, argues that immersive media itself does not necessarily improve learning [28]. Therefore, providing systematic or structured content and process design through learning/teaching theories is of great importance and will help to carry out curriculum/teaching/learning design in a scientific manner. In order to facilitate the new knowledge creation process of users, instructional goals supported by certain principles are recommended by CTML [29], and extraneous cognitive load can be reduced by applying five principles: Coherence, Signaling, Redundancy, Redundancy, and Temporal Contiguity principles [30]. The design of this research was developed on the basis of the theoretical framework of CTML.

### 2.2. Research Model and Hypotheses

**Figure 1:**
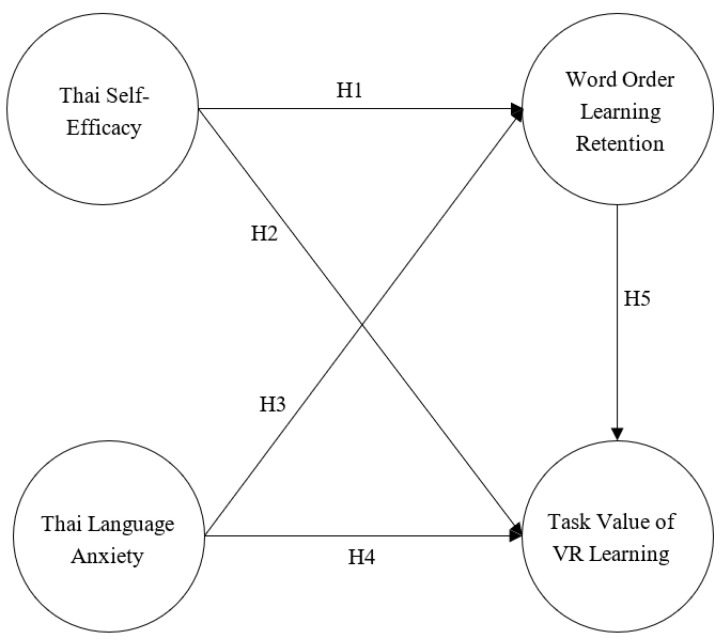
Research Model.

CTML holds that learning outcomes are subject to various cognitive factors in the context of multimedia learning. Thus, in a multimedia-based learning field, positive cognitive factors will facilitate learners’ learning performance and positive experience, and vice versa. Accordingly, a research model was designed based on CTML, and the correlates between Thai self-efficacy, Thai language anxiety, word order learning retention, and task value of learning through VR were hypothesized, as shown in Figure 1.

### 2.3. Research Hypotheses

#### 2.3.1. Relationship between Thai Self-Efficacy and Word Order Learning Retention

Banduraargues that a learner’s self-efficacy results from the complex interplay between their cognition, behavior, environment, and other factors [31]. The beliefs that learners hold about their abilities affect the efforts and persistence involved in undertaking tasks or activities, as well as the expected outcome of completing the tasks [32]. Individuals with stronger self-efficacy are likely to face difficulties, engage in tasks or activities, and achieve better performance later [33]. One of the important aspects of assessing learning performance is retention, and deep learning occurs when learners manage to transfer what they have learned to a new situation and are able to demonstrate that they really understand through inferring and generalizing learned concepts or processes [34]. Challenges include that some learners quickly forget the content acquired in school and fail to recall the knowledge later when they need it [35]. Studies also show that learners’ active participation in the content contributes to better understanding. It is assumed to enhance the retention of knowledge [36,37]. Thus, the relationship between participants’ Thai self-efficacy and retention of word order learning is hypothesized as follows:

**H1.**  *Thai self-efficacy is positively related to word order learning retention*.

#### 2.3.2. Relationship between Thai Self-Efficacy and Task Value of VR Learning

The concept of task value comes from Eccles et al.’s expectancy-value theory; it refers to the value that individuals perceive when learning, determining their further learning plans [38]. Self-efficacy is about people’s beliefs in their capabilities to exert influence over events important to their lives. People are willing to face difficulties or persist when they believe they can produce expected outcomes through their deeds [39]. Scholars have also found that self-efficacy was strongly related to academic outcome expectations [40,41]. The research of Hong et al. pointed out that self-efficacy was significantly positively related to the increase in learners’ cognition, affection, behavioral engagement, and participation value [42]. However, related research on Thai learning is limited, and although self-efficacy has been shown by many studies to have positive indirect effects on task value, whether Thai self-efficacy has a direct influence on task value in VR learning environments is yet to be confirmed.

How participants’ Thai self-efficacy is related to their task value of VR learning was hypothesized as follows:

**H2.**  *Thai self-efficacy is positively related to the task value of VR learning*.

#### 2.3.3. Relationship between Thai Language Anxiety and Word Order Learning Retention

Horwitz et al. defined foreign language anxiety as “a distinct complex of self-perceptions, beliefs, feelings, and behaviors related to classroom language learning arising from the uniqueness of the language learning process” [43]. Learners’ perceived language anxiety may have a negative impact on their language learning [44]. The anxiety that learners feel when learning a language generally stems from the classroom environments, difficulties concerning oral and reading, listening performance, and the anxiety reaction often prevents them from performing successfully in foreign languages [45,46,47]. Additionally, compared to repeated study, testing is a more beneficial learning activity for learners to remember information and is more effective in terms of retaining knowledge. However, excessive tests also induce anxiety that disrupts learning [48]. Therefore, regular testing is proposed to prevent a high level of test anxiety [49,50]. In this research, participants were asked to take part in quiz games via a “Multilingual VR Learning Assessment System” to assist learning; hence the relationship between Thai language anxiety and word order learning retention was hypothesized as follows:

**H3.**  *Thai language anxiety is negatively related to word order learning retention*.

#### 2.3.4. Relationship between Thai Language Anxiety and Task Value of VR Learning

Task value, in the expectancy-value theory, refers to the degree to which learners consider that the academic task is worth completing [51]. In addition to the value, students’ level of anxiety during learning, either high or low, also has varying degrees of impact on their learning motivation, learning attitudes, learning achievement, and language learning processes [52,53]. Students may even exhibit avoiding behaviors (like missing class) when they have negative feelings toward or have an aversion to learning foreign languages [54]. Studies have also found that females experience more anxiety than males when learning foreign languages [55,56]. Saito and Samimy’s investigation into the effect of foreign language anxiety on learners’ performance confirmed that a high level of language anxiety does indeed have a negative impact on learners’ performance and may decrease their self-confidence and interest in foreign language learning [57]. The relationship between participants’ Thai language anxiety and the task value of learning through VR was hypothesized as follows:

**H4.**  *Thai language anxiety is negatively related to the task value of VR learning*.

#### 2.3.5. Relationship between Word Order Learning Retention and Task Value of VR Learning

Forgetting happens, no matter what age group or background learners are from. Teachers often focus their efforts on helping students acquire new knowledge and skills, but newly acquired information is vulnerable and easily slips away. Teachers often focus on helping learners acquire new knowledge and skills but seldom on helping to retain them [58]. Hintzman argues that repetition is viewed to be a powerful way to enhance memory [59]. Further, Cepeda et al. found that spaced learning is beneficial to the retention of learned items [60]; and compared with mass learning, paced learning can significantly improve long-term retention of knowledge or skills [61]. Gee and Coyne addressed that computer games help students improve memory, concentration, and skills like critical thinking [62,63]. Studies have also found that game-based learning effectively improves retention [64]. Hong et al. noted that in addition to clearly defined goals and rules of learning [65], what is important about computer game-based learning is that the game itself must be fun enough to attract students so that they gain learning value from it, and the computer games should also be able to improve students’ performance and reduce the complexity in the learning process [66]. The relationship between Thai word order learning retention and participants’ perceived task value of learning through VR was thus hypothesized as follows:

**H5.**  *Word order learning retention rate is positively related to the task value of VR learning*.

### 2.4. Procedure

The participants of this research were students from three advanced Thai classes, and the experiment lasted for 10 weeks in one semester. In the first week, the researcher first identified himself and introduced the purpose of this research, and clearly expressed that this experimental research would only be carried out with the consent of the students. After signing the informed consent form, students were then given a pre-test with 150 Thai sentences for word order practice, and the test time was 90 min. 

**Figure 2:**
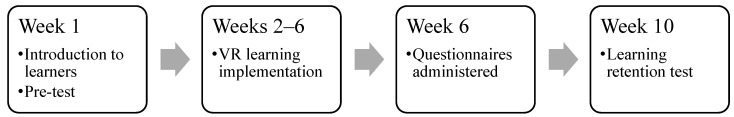
Experiment Procedure.

In the second week, the researcher provided correct answers to the 150 questions so that the participants would learn where they had answered correctly and incorrectly. After that, 10 min were given so that the subjects could familiarize themselves with the operation of the “Multilingual VR system,” a language learning game in which participants are immersed in a three-dimensional virtual environment to practice Thai word order through VR technology. The participants then began the practice using the system. The practice time was about 20 min for 5 consecutive weeks (weeks 2–6). The 50 questions that most students got wrong were then selected out of the 150 questions in the pre-test. They would appear randomly in the game. In the sixth week, after the exercise, questionnaires were distributed for the participants to fill out. In the 10th week, a word order learning retention test was conducted as an assessment of the word order learning retention, as shown in Figure 2.

### 2.5. Participants

The research adopted a single-group quasi-experiment, and the participants were selected via purposive sampling, a sampling method to be used when the sample is small because the researcher has specific requirements for the sample and needs full cooperation from the chosen subjects, according to Panacek and Thompson [67]. Therefore, considering the small number of Thai language learners and the limited number of accredited academic institutions that deliver Thai courses on a regular basis, this study adopted purposive sampling, selecting a total of 90 participants from three advanced Thai language classes at a university in northern Taiwan, with 30 students per class. Most of the participants were Taiwanese adults aged 20–60. They had studied Thai for an average of 1–3 years and had completed at least Thai Basics 1, Thai Basics 2, and Thai Basics 3. Of the 90 participants (returned questionnaires), six invalid data were removed, resulting in a total of 84 valid participants, with an effective return rate of 93.3%, of whom 30 were males and 54 females. Among the participants, 13 (15.5%) had a junior college diploma or below, 51 (60.7%) had a bachelor’s degree, and 20 (23.8%) had a master’s degree or higher. Regarding their experience in learning Thai, 14 (17.7%) had 9 to 12 months of experience, 16 (19.1%) had 12 to 18 months of experience, 31 (36.9%) had 19 months to 2 years of experience, and 23 (27.4%) had more than 2 years of experience. None of the participants in this study had previous experience with VR-assisted learning.

### 2.6. Teaching Tools

**Figure 3:**
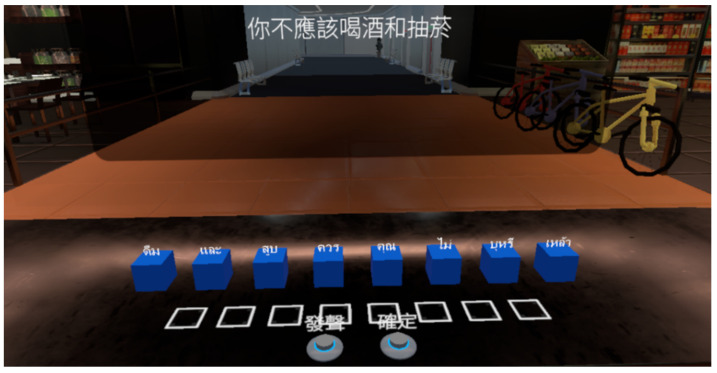
Game Screenshot. Gameplay Screenshots. 你不應該抽菸和喝酒 You should not be smoking or drinking alcohol. 發聲 Sound 確認 Confirmation.

**Figure 4:**
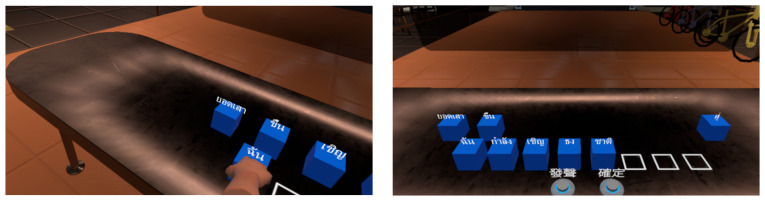
Gameplay Screenshots. 你不應該抽菸和喝酒 You should not be smoking or drinking alcohol. 發聲 Sound 確認 Confirmation.

**Figure 5:**
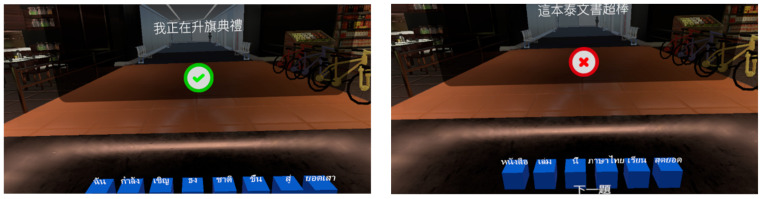
Answer Result Screenshots. Gameplay Screenshots. 你不應該抽菸和喝酒 You should not be smoking or drinking alcohol. 發聲 Sound 確認 Confirmation.

The benefits of learning via VR tools are based on the theory of embodied cognition. The theory posits that one’s embodied experiences are necessary for the formation and establishment of cognition [68]. Therefore, this study adopts an embodied VR system, the “Multi-language VR Learning Assessment System,” developed by the Digital Learning Laboratory of National Taiwan Normal University (NTNU). This VR system is a commercialized educational platform, of which access rights can be purchased from NTNU. The advantage of this system is that it allows teachers to freely fill in the learning content (question bank) in the game, divide levels according to difficulty, and switch target languages (e.g., native Chinese speakers learning Thai or native Thai speakers learning Chinese, etc.). All usage history is recorded in databases on the internet. System screenshots are shown in Figure 3, Figure 4 and Figure 5 below. After logging in, participants would first select the topic and the language used. For example, if their native language is Chinese, the topic language should be “Chinese,” as shown in Figure 3 and Figure 4.

### 2.7. Measurement

This research was based on quantification-based validation and collected data through a questionnaire survey. Items in the questionnaire were adapted from past studies, as described below, and reviewed by experts to ensure validity. A 5-point Likert scale was used for responses, ranging from 1 (strongly disagree) to 5 (strongly agree).

#### 2.7.1. Thai Self-Efficacy

The questionnaire items to measure participants’ perceptions of Thai language learning were modified from the self-efficacy scale from Watthanapas et al. [69]. Examples include: “I believe that I can learn Thai well as long as I do more practice” and “I can find out my own way to learn Thai well by myself”.

#### 2.7.2. Thai Language Anxiety

The Language Anxiety Scale was adapted from Hwang et al. and Horwitz et al. to measure participants’ perceptions of Thai language anxiety [45,70]. Examples include: “I feel nervous when I can’t describe what I know in Thai” and “The course progress in my class is so fast that I’m worried that I won’t be able to keep up because of my insufficient proficiency in Thai”.

#### 2.7.3. Word Order Learning Retention

The word order learning retention measurement in this study refers to a test on the 50 sentences after the experiment. The 50 questions were selected out of the 150 questions in the pre-test on Thai word order. These 150 questions came from the teaching materials of advanced Thai language courses at the continuing education center at a national university in northern Taiwan. The grammar rules and sentence patterns of these 150 questions in the pre-test of Thai word order were those that most Taiwanese students found difficult to learn. After the pre-test, 50 questions that most students got wrong were selected and imported into the “Multi-language VR Learning Assessment System” for Thai word order practice for five consecutive weeks. After four more weeks, students were tested with these 50 questions again as a post-test for word order learning retention.

#### 2.7.4. Task Value of VR Learning

This measurement was revised from the learning value scale of Chang and Jan and Hwang et al. to measure participants’ perceived task value of the VR Thai word order learning system [71,72]. Item examples are, “I think this VR system allows me to learn about Thai sentence structure more easily” and, “I think this VR system is a good tool suitable for repeated practice of the knowledge I have learned”.

### 2.8. Statistical Methods

In this study, partial least squares (PLS) analysis was used to validate the path relations and to analyze the factor loadings (FL) and paths among constructs. PLS is an alternative to the covariance SEM method for estimating structural equation models [73]. PLS works well in estimating complex models with small sample sizes [74]. Furthermore, PLS-SEM is one of the appropriate methods to evaluate path coefficients in structural models [75]. According to Ramdhani and Ramdhani, the research model verification methodology is a concept based on a literature review [76]. It observes the suitability of the research model following criteria and takes steps to obtain a description of whether the research model matches the system it represents. Therefore, it is an appropriate tool for this research.

## 3. Results and Discussion

### 3.1. Item Analysis

This research applied first-order confirmatory factor analysis for item analysis. Scholars suggest that the threshold of χ^2^/*df* be less than 5, RMSEA be less than 0.10, GFI and AGFI be higher than 0.80, and items with factor loadings (FL) not higher than 0.50 be removed from the original questionnaire [77,78]. Results are shown in Table 1. Thus, the number of items for Thai self-efficacy was reduced from eight to five, for Thai language anxiety from 12 to seven, and for task value of VR learning from six to five.

The external validity of the items was used to judge the extent of the study interpretation [79]. A *t*-test was performed on the respondents in the top 27% and bottom 27% scoring groups; if the *t* value (critical ratio) exceeds 3 (*** *p* < 0.001), the external validity can be considered significant. Table 2 shows that the *t*-values range from 5.10 to 16.41 (*** *p* < 0.001), indicating that all items in this research were discriminative. That is, they could differentiate the responses of different samples [80].

### 3.2. Reliability and Validity Analysis

In this research, Cronbach’s α was conducted to examine the internal consistency in the scale items, and composite reliability (CR) was conducted to re-examine reliability. Hair et al. suggested that Cronbach’s α and CR values be higher than 0.70 to be considered an acceptable result [77]. The Cronbach’s α value ranged from 0.79 to 0.91, and the CR values from 0.85 to 0.93, meeting the suggested criteria, as shown in Table 2.

Convergent validity is judged by factor loading (FL) and average variance extracted (AVE). Hair et al. pointed out that the FL value should be higher than 0.50, or the items should be removed [77]. All the items retained in the research met the suggested standard: the FL value ranged from 0.73 to 0.80, as shown in Table 2. Hair et al. suggested that the AVE value should be greater than 0.50 to indicate good convergent validity for each construct [74]. All AVE values were between 0.54 and 0.65, as shown in Table 2.

Awang proposed that the AVE square root of each construct should be higher than the Pearson correlation coefficient of other constructs to indicate that the construct has discriminant validity [81]. The analysis results showed that each construct in this research had discriminant validity, as shown in Table 3.

### 3.3. Analysis of the Word Order Learning Retention

The results of the descriptive analysis of word order learning retention showed that the mean of the participants’ word order learning retention was 41.26, the standard deviation was 5.944, and the median was 42, as shown in Table 4.

### 3.4. Analysis of Research Model Validation

**Figure 6:**
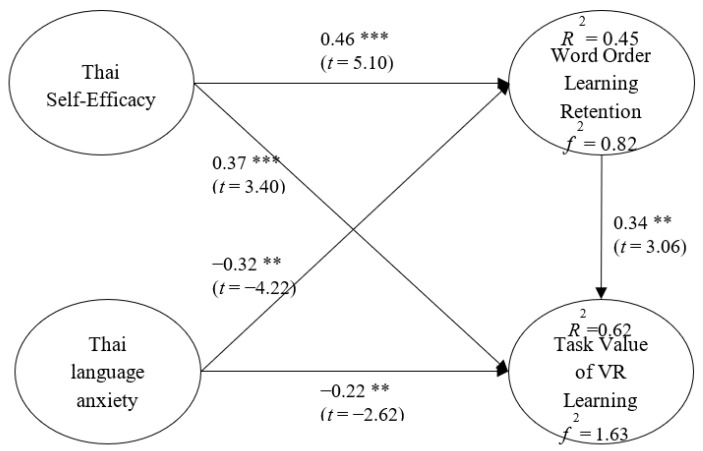
Research Model Validation. *t* = 2.57 = ** *p* < 0.1; *t* = 3.29 = *** *p* < 0.001.

Thai self-efficacy was positively related to word order learning retention (β = 0.46 ***; *t* = 5.1); Thai self-efficacy was positively related to task value of VR learning (β = 0.37 ***; *t* = 3.4); Thai language anxiety was negatively related to word order learning retention (β = −0.32; *t* = −4.22); Thai language anxiety was negatively related to task value of VR learning (β = −22; *t* = −2.62); and word order learning retention was positively related to task value of VR learning (β = 0.34 **; *t* = 3.06), as shown in Figure 6. The explanatory power of Thai self-efficacy and language anxiety on word order learning retention was 45%, and f2 was 0.82; the explanatory power of Thai self-efficacy, language anxiety, and word order learning retention on task value of VR learning was 62%, and f2 was 1.62, respectively, as shown in Figure 6.

### 3.5. Discussion

#### 3.5.1. Thai Self-Efficacy Was Positively Related to the Retention of Word Order Learning

A learner’s beliefs about his or her abilities influence the effort and perseverance with which he or she participates in a task or activity, as well as the expected outcome of completing the task [32,82]. Gao et al. found that people with high self-efficacy are more likely to accept challenges and devote themselves to tasks or activities, and usually have better achievements [33]. Self-efficacy is a critical affective construct that has a significant influence on the success and retention of students’ learning [83]. Students with higher academic self-efficacy tend to set higher academic goals, and academic self-efficacy may be the most powerful predictor of students’ academic achievement and retention [84].

The results of this research showed that learners’ Thai self-efficacy was positively related to learning retention, in line with the above-mentioned research results. In the two constructs, the mean of Thai self-efficacy was 4.043 (*SD* = 0.446), and the average score of each item ranged from 3.92 to 4.30, indicating the participants have a positive perception of Thai self-efficacy; and the average score of learning retention was 41.26 (*SD* = 5.944), meaning that the participants’ learning of Thai word order through the use of VR was well retained. The results revealed that if learners had a higher level of self-efficacy, they would achieve better retention by learning Thai word order through VR.

#### 3.5.2. Thai Self-Efficacy Was Positively Related to Task Value of VR Learning

Self-efficacy refers to one’s self-assessment of one’s capacity to perform specific activities. Students with a stronger sense of self-efficacy put more effort into the completion of their academic tasks [85], and their self-regulated learning and competence in writing can be improved [86].

Prior research finds that self-efficacy is positively related to task value [87], and that self-efficacy and task value are positively correlated with achievement behaviors [88]. Both task value and self-efficacy have a significant influence on learning engagement [89,90]. Moreover, self-efficacy and task value play an important role in promoting learners’ achievement-related behaviors: choice, effort, persistence, and continuation [88], and higher self-efficacy and task value relate to better task performances [91].

The result of this research shows that learners’ Thai self-efficacy was positively related to task value, lending support to the aforementioned research results. In these two constructs, the mean of Thai self-efficacy was 4.043 (*SD* = 0.446), and the average score of each item ranged from 3.92 to 4.30, representing participants’ positive perceptions of Thai self-efficacy. The mean task value is 3.645 (*SD* = 0.590), indicating that participants perceived the positive value of learning Thai word order in the VR environment. Accordingly, learners with a higher level of self-efficacy attain higher task value belief in learning Thai word order through VR.

#### 3.5.3. Thai Language Anxiety Was Negatively Related to the Retention of Word Order Learning

Horwitz et al. defined foreign language anxiety as a complicated mix of self-perceptions, beliefs, feelings, and behaviors which occur in the language learning process, and many studies argue that language anxiety has a negative impact on learners [45]. The anxiety that learners feel when learning a language stems from the classroom environment generally and difficulties concerning oral, reading, and listening performance [45,46,47]. The anxiety reaction often prevents learners from performing successfully in foreign languages. In addition, learners feel anxious about making mistakes and being corrected by teachers in class, as well as nervous about failing exams [92]. Excessive testing triggers test anxiety, which further impedes learning [48].

Many studies have confirmed that game-based learning can effectively enhance students’ learning motivation, and, if appropriately integrated with learning strategies, can effectively improve learners’ performance and retention [93]. However, the direct correlation between language anxiety and learning retention has not been fully examined. The results of this research exhibited that learners’ Thai language anxiety was negatively related to their learning retention. In the two constructs, the mean of Thai language anxiety is 2.961 (*SD* = 0.678), and the average score of each item was between 3.2 and 2.73, signifying that the participants perceived a middle to low level of language anxiety. The average score of learning retention was 41.26 (*SD* = 5.944), meaning that participants had good retention of Thai word order by learning through VR. The analysis results implied that learners with a lower level of language anxiety had better retention of Thai word order learned through VR.

#### 3.5.4. Thai Language Anxiety Was Negatively Related to Task Value of VR Learning

Language anxiety, a very common phenomenon in foreign language learning, refers to learners’ upsetting feeling of being unable to be themselves and to genuinely connect with others through their limited competence in a new language [94], or learners’ sense of fear or worry which arises in the face of certain situations in foreign language learning [95]. Foreign language learning is affected by individual differences such as aptitude, motivation, beliefs, anxiety, and personality [96]. Students’ levels of anxiety during learning, high or low, have varying degrees of impact on their learning motivation, learning attitudes, learning achievement, and language learning processes [52,53]. Language anxiety is demonstrated in empirical studies to be detrimental to foreign language learning and its use [97].

Task value refers to the degree to which learners consider that the academic task is worth completing [51]. Task values can serve as a predictor of learners’ future course plans and enrollment decisions [38]. The findings of this research showed that learners’ Thai language anxiety was negatively related to task value. The mean of Thai language anxiety was 2.961 (*SD* = 0.678), and the average scores of each item were between 3.2 and 2.73, representing that the participants’ Thai language anxiety was mild; and the mean of task value was 3.645 (*SD* = 0.590), indicating that participants’ perception of the task value of Thai word order via VR was positive. In other words, learners with a lower level of language anxiety perceive a higher level of task value when learning Thai word order through VR.

#### 3.5.5. The Retention of Word Order Learning Was Positively Related to Task Value of VR Learning

Cepeda et al. found that spaced learning is beneficial to the retention of learned items [60]. Compared with mass learning, paced learning can significantly improve the retention of knowledge or skills [61]. In addition, Gee and Coyne argue that computer games help students improve memory, concentration, and skills such as critical thinking [62,63]. Studies have also found that game-based learning effectively enhances students’ learning motivation, and, if appropriately integrated with learning strategies, can effectively improve learners’ performance and retention [64,93].

The value of integrating alternative activities (games, exercises, and simulations) and environments is recognized as they help to stimulate student interest in the learning environment, increase student understanding, and improve learned retention with meaningful repetition [98]. A finding of this research is that the learner’s learning retention is positively related to task value, which supports previous research results. The mean of learning retention was 41.26 (*SD* = 5.944), and the median of the items was 42, implying that participants’ learning of Thai word order through VR yielded profound retention; the mean of task value was 3.645 (*SD* = 0.590), indicating that the participants using VR to learn Thai word order perceived good task value. Based on the findings, learners with a higher level of learning retention had better-perceived value of learning through VR.

## 4. Conclusions and Recommendations

### 4.1. Conclusions

The results of this research showed that Thai learners’ Thai self-efficacy was positively related to word order learning retention, and Thai self-efficacy was positively related to the task value of VR. It can be seen from the descriptive analysis that the learners indeed had a high level of Thai self-efficacy, and their perception of task value in VR learning environments was also positive.

Meanwhile, Thai language anxiety showed a negative correlation with the retention of Thai word order learning as well as VR task value. The analysis inferred that these Thai learners reported moderate perceptions of language anxiety, performed well in word order learning retention, and had a high level of the perceived value of learning through VR.

In the past, research on game-based language learning was mostly about its effect on English vocabulary learning, English writing learning, or its application to English learning strategies, but seldom to the learning of word order. This research, based on CTML and the theory of embodied cognition, is one of the first studies to investigate the effectiveness of applying a VR system to Thai word order learning. From the results of this research, it can be observed that the CTML can well explain Thai learners’ affective and cognitive states regarding the use of VR and their learning outcomes. The examined results of this research may help to extend the explanatory power of CTML to studies on the application of VR in language learning.

Furthermore, the findings show that Thai language learners perceived the value of learning through VR, recognizing its benefit to their learning performance as they could be entirely immersed in the learning game and practice Thai word order repeatedly with body movement at the same time. This suggests that the theories of embodied cognition (TEC) can adequately explain the effect of VR learning on the improvement of Thai learners’ memory and performance. The examination results of this research are of help to strengthen the ability of the TEC to explain how applying VR in foreign language learning can help learners to enhance the retention of knowledge.

### 4.2. Recommendations

In the era of the metaverse, the digital transformation of education will be even faster. Although VR is not yet widely used in classrooms, numerous studies, including this study, have found its educational value. Therefore, emerging technologies, including VR, will play an important role in language education in the near future. This study suggests that language teachers try to utilize VR as an auxiliary or primary teaching tool to help language learners obtain learner-centered learning experiences. Immersive VR can also provide learners with more realistic learning situations, which will help them engage in the learning environment.

In addition, this study also found that language anxiety was negatively correlated with learning retention and value experience. How to effectively reduce language anxiety is thus an important goal. This study also suggests that language teachers, either through investigation or observation, identify and help learners with high language anxiety develop learning strategies or methods to relieve anxiety and enhance their belief in competence in order for them to achieve good learning results.

Still, as the popularity of VR is low, it is particularly important for teachers and students to be trained before using it. If students or teachers are not familiar with educational technology, they will not be able to appreciate it or even be willing to use it, even if it can bring good educational results. Although in this study, no participant was reported to have difficulties adapting to the VR environment and experience discomfort, past studies did find some users unable to adapt to immersive devices. Teachers who intend to use VR as an auxiliary learning tool are recommended to also prepare alternative learning plans or emergency measures in case of situations where students experience dizziness or other unadaptable reactions.

### 4.3. Limitations and Future Study

In this study, learning retention was considered an indicator of the learning effect of VR. As participants were still attending Thai language classes prior to taking the tests, their learning retention might have been affected by continued learning. Therefore, in future research, interviews can be conducted to gain insight into participants’ views on the impact of VR on learning effectiveness or retention so as to gain a more comprehensive understanding of the effectiveness of VR on language learning. This will also help to understand the differences between participants’ past experiences with applications and their experiences with VR. Other than that, the findings of this research confirm that learners using VR to learn Thai word order achieved positive learning outcomes. The use of VR technology as a learning tool allows learners to experience more liveliness and interactivity in the learning process, and contributes to alleviating the boredom of repetitive studying in the classroom. This research focused mainly on the effectiveness of applying a VR language learning system to the practice of Thai word order in sentences. It has not yet been able to investigate the effect of the VR language learning system on Thai vocabulary, Thai alphabet and spelling, Thai pronunciation practice, Thai listening, or other language skills. Therefore, future studies may consider examining how a VR language learning system can be utilized to learn other skills related to the Thai language and how it can be integrated into learning and instruction strategies.

Since the outbreak of COVID-19 in 2020, many countries have been using edtech (educational technologies) of all sorts, for example, online learning, online video platforms, mobile apps, and virtual platforms, to support access to remote learning [99], and Taiwan is no exception. Learners had to suddenly adjust to distance learning and gradually come to accept technology-assisted learning. Therefore, it is suggested that future educators incorporate advanced edtechs like VR with learning tasks of other language skills such as alphabets, pronunciation, and vocabulary. Also, how the design of VR learning systems can be improved (like taking into account interactive functions such as speech recognition to facilitate learning in conversation practice, cultural learning, etc.) is yet another field to be explored.

## Figures and Tables

**Table 1:** First-order Confirmatory Analysis.

Overall Model Fit	Value	Thai Self-Efficacy	Thai Language Anxiety	Task Value of VR Learning
χ^2^	-	5.1	23	7.4
*df*	-	5	14	5
χ^2^/*df*	<5	1.02	1.64	1.48
RMSEA	<0.10	0.01	0.09	0.08
GFI	>0.80	0.98	0.93	0.97
AGFI	>0.80	0.93	0.86	0.90
FL	>0.50	0.67~0.78	0.70~0.86	0.58~0.90
*t*	>3	5.10~9.34	6.76~16.41	6.60~12.72

**Table 2:** Reliability and Validity Analysis.

Construct	M	SD	α	CR	AVE	FL
	-	-	>0.70	>0.70	>0.50	>0.50
Thai Self-Efficacy	4.04	0.45	0.79	0.85	0.54	0.73
Thai Language Anxiety	2.96	0.68	0.91	0.93	0.65	0.80
Task Value of VR Learning	3.65	0.59	0.89	0.90	0.65	0.80

**Table 3:** Discriminant Validity.

Constructs	1	2	3	4
1. Thai Self-Efficacy	(0.85)			
2. Thai language anxiety	−0.49	(0.89)		
3. Thai Word Order Learning Retention	0.62	−0.54	(1)	
4. Task Value of VR Learning	0.65	−0.58	0.67	(0.89)

Note: The values on the diagonal are the square root of the AVE values, and the other values are the correlation coefficient values.

**Table 4:** Analysis of Word Order Learning Retention.

Construct	M	*SD*	Md
Word Order Learning Retention	41.26	5.944	42

## Data Availability

The original contributions presented in the study are included in the article. Further inquiries can be directed to the corresponding author.
